# Local structure of Amorphous carbon investigated by X-ray total scattering and RMC modeling

**DOI:** 10.1038/s41598-024-76796-x

**Published:** 2024-10-25

**Authors:** Masatsugu Yoshimoto, Kazuki Ito, Kazuhiko Omote

**Affiliations:** X-ray Research Laboratory, Rigaku Corporation, 3-9-12 Matsubara-cho, Akishima, Tokyo, 196-8666 Japan

**Keywords:** Reverse Monte-Carlo (RMC) modeling, Total scattering, Persistent homology, Amorphous carbon, Chemistry, Materials science, Mathematics and computing, Nanoscience and technology

## Abstract

Amorphous carbon is a promising candidate as an energy storage material. In this paper, we performed an X-ray total scattering measurement, RMC modeling, and persistent homology analysis for amorphous carbon samples fabricated at two different heat treatment temperatures. According to the analysis of the nearest-neighbor carbon atoms and their angular histogram, the sample treated at higher temperature shows higher connectivity between carbon atoms than that treated at lower temperature. Furthermore, topological data analysis (persistent homology, PH) reveals quantitative results that relate ring structure and the connectivity between carbon atoms.

**Supplementary Information:**

The online version contains supplementary material available at 10.1038/s41598-024-76796-x.

## Introduction

Amorphous carbon, which contains mesopores in its structure, is used in various fields, including catalytic support for fuel cells^1–5^, anode material for Li-ion batteries^6–10^, energy storage^11–13^, etc. There are several well-known methods to form the mesopores into amorphous carbon, including activated and template methods^14–18^. Mesoporous carbon that uses magnesium oxide MgO as a template^19–21^ has several advantages. First, it is easy to control the mesopore size. Next, MgO can be easily eliminated from the mesoporous carbon material by a weak acid. Lastly, MgO can be reused as a template material.

It is widely known that there are the three reaction stages to change amorphous carbon to graphite^22^. First, an amorphous carbon of mixed sp^2^ and sp^3^ structure is formed from the precursor molecule below *T* = 1000 °C. Next, a network structure develops in the amorphous carbon around *T* = 1500 °C. Finally, graphitization occurs above *T* = 2200 °C. Although there have been many reports on the systematic study of local structural changes in amorphous carbon with heat treatment by X-ray diffraction^23,24^ and Raman spectroscopy^25,26^, the local atomic arrangements in the amorphous carbon are not directly discussed. Moreover, many researchers have studied the atomic-scale structure of amorphous carbon using molecular dynamics (MD)^27,28^ and the reverse Monte-Carlo (RMC) method^29–31^. However, there is little research focusing on the carbon local structure formed among carbon atoms because these researchers have been mainly interested in the character of the pores (e.g., pore connectivity, the amount of gas storage available in the pores, the pore size distribution etc.).

X-ray total scattering measurement is the most suitable analysis to evaluate the local structure in matter, and it can be applied to many fields: crystalline materials^32^, glasses^33^, amorphous solids^34^ and liquids^35^. The experimental pair distribution function PDF *G*(*r*) is directly obtained from the Fourier transform of the observed structure factor *S*(*Q*)^36^ as follows:

1 $$\begin{aligned}G\left(r\right)=\frac{2}{\pi}\int_{{Q}_{\text{min}}}^{{Q}_{\text{Max}}}Q\left[S(Q)-1\right]\text{sin}\:Qr\:dQ,\end{aligned}$$

where *Q* is the scattering vector defined as $\:Q=4\pi\:\text{sin}\theta\:/\lambda\:$, where $\:\theta\:$ and $\:\lambda\:$ are the scattering angle and the wavelength of the X-rays, respectively. The experimental PDF only provides qualitative local structure because it only expresses one-dimensional information; average bond lengths and the distribution of bond lengths. To investigate quantitative three-dimensional local structure based on experimental data, RMC modeling combined with an X-ray total scattering measurement is the most effective method. This approach can construct a plausible structural model by minimizing the difference between the experimental and calculated scattering functions.

Recently, persistent homology (PH) analysis has attracted interest to reveal the relationship between the atomic-scale structure and characteristic properties of a material^37–42^. PH analysis provides us with multiscale properties including not only short-range but also mesoscale from the simulated structural model.

The filtration process for PH analysis in a three-dimensional system is as follows. First, spheres with radius *r* are placed at each atom. Next, *r* is increased gradually. The process of increasing the radius is referred to as *time*. Consequently, the spheres begin to intersect, and then edges are set between the centers of the intersecting spheres with advancing time. When the edges form a closed ring, *r* is recorded as a *birth* time. Finally, as the radius increases, a closed ring disappears. The radius where the ring disappears is recorded as a *death* time. As a PH analysis result, we obtain the two-dimensional persistent diagram (PD) that visualizes the birth-death pair of each ring. Generally, two types of PD are classified when PH analysis is applied to a 3D structural model. One is one-dimensional PD (PD1), which relates the ring and hole properties in the simulated structure. The other is two-dimensional PD (PD2) that relates the pore and cavity in the simulated model. We focus on PD1 to evaluate the ring structure between carbon atoms in this work.

In this study, we perform the X-ray total scattering measurement and RMC modeling on amorphous carbon samples fabricated at two different annealing temperatures. In order to investigate the relationship between the local structure of amorphous carbon and the annealing temperature, we apply a detailed analysis to the constructed structural model; the angular histogram, the histogram of the nearest-neighbor coordination numbers, and persistent homology analysis. Then, we will discuss the nearest-neighbor atomic arrangement of the obtained structure and the network structure extracted by PD1 analysis.

## Results and discussion

**Fig. 1:**
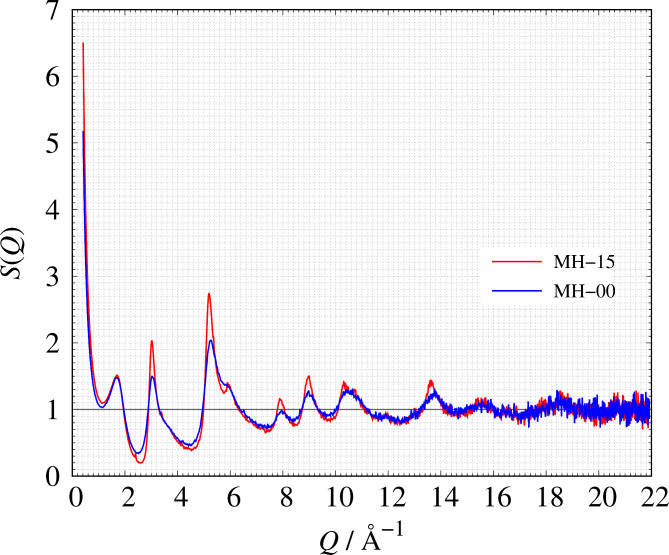
The observed structure factor *S*(*Q*): red solid line: MH-15, blue solid line: MH-00.

**Fig. 2:**
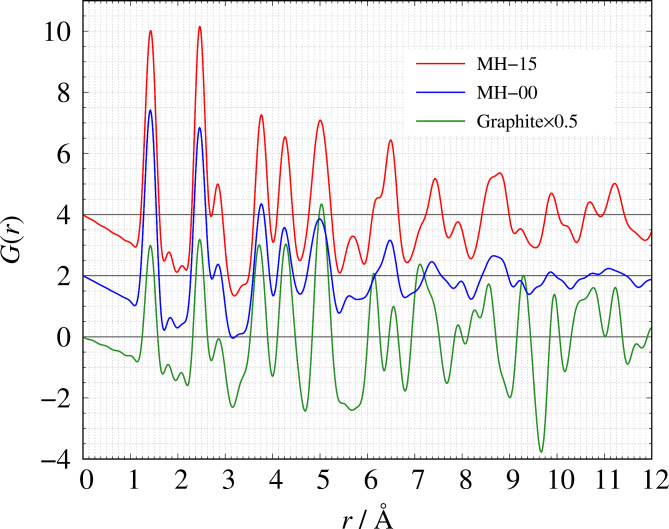
The observed pair distribution function *G*(*r*): red solid line: MH-15, blue solid line: MH-00, green solid line: graphite.

Detailed sample information about the two amorphous carbons can be found in the Methods section. Figure 1 shows the structure factors *S*(*Q*) of MH-15 and MH-00. MH-15 has a carbon arrangement with greater long-range order than MH-00 because the fringe of MH-15 appearing at *Q* > 2 Å^−1^, which relates to local atomic structure, is higher than that of MH-00. Despite the heat treatment temperature differences between MH-15 and MH-00, the peak at *Q* = 1.7 Å^−1^ shows almost the same intensity and peak width. The result suggests that the peak is related to the mesoscale structure of the precursor but not the interlayer distance of graphene sheets, whose peak appears between 1.85 Å^−1^ < *Q* < 1.88 Å^−1^ shown in Fig. S1. *T* = 1500 °C is too low of a temperature for graphitization to occur. At *r* < 3.0 Å, the local carbon arrangement of MH-15 and MH-00 is almost the same graphitic 6-member ring structure shown in Fig. 2. It is known that Archimedes’ principle is difficult to use to measure atomic-scale density of porous materials because liquid or gas molecules can’t penetrate some mesopores. In the previous study, we showed that the atomic-scale density can be estimated from the experimental *S*(*Q*) with an accuracy of ± 5% for both amorphous and crystalline materials^43^. This means that *S*(*Q*) is sensitive to the atomic density and the proper number of atoms must be introduced into the simulation box to construct a reasonable RMC model. We have estimated atomic densities of MH-15 and MH-00 from the observed *S*(*Q*) shown in Fig. 1. The estimated atomic densities of those samples and numbers of carbon atom in the simulation box are listed in Table 1.

**Table 1:** The RMC calculation conditions of MH-15 and MH-00; atomic number density and the number of atoms.

Sample	Atomic number density *r* (Å^−3^)	The number of atoms in the calculation box
MH-15	0.0767	4910
MH-00	0.0626	4011

**Fig. 3:**
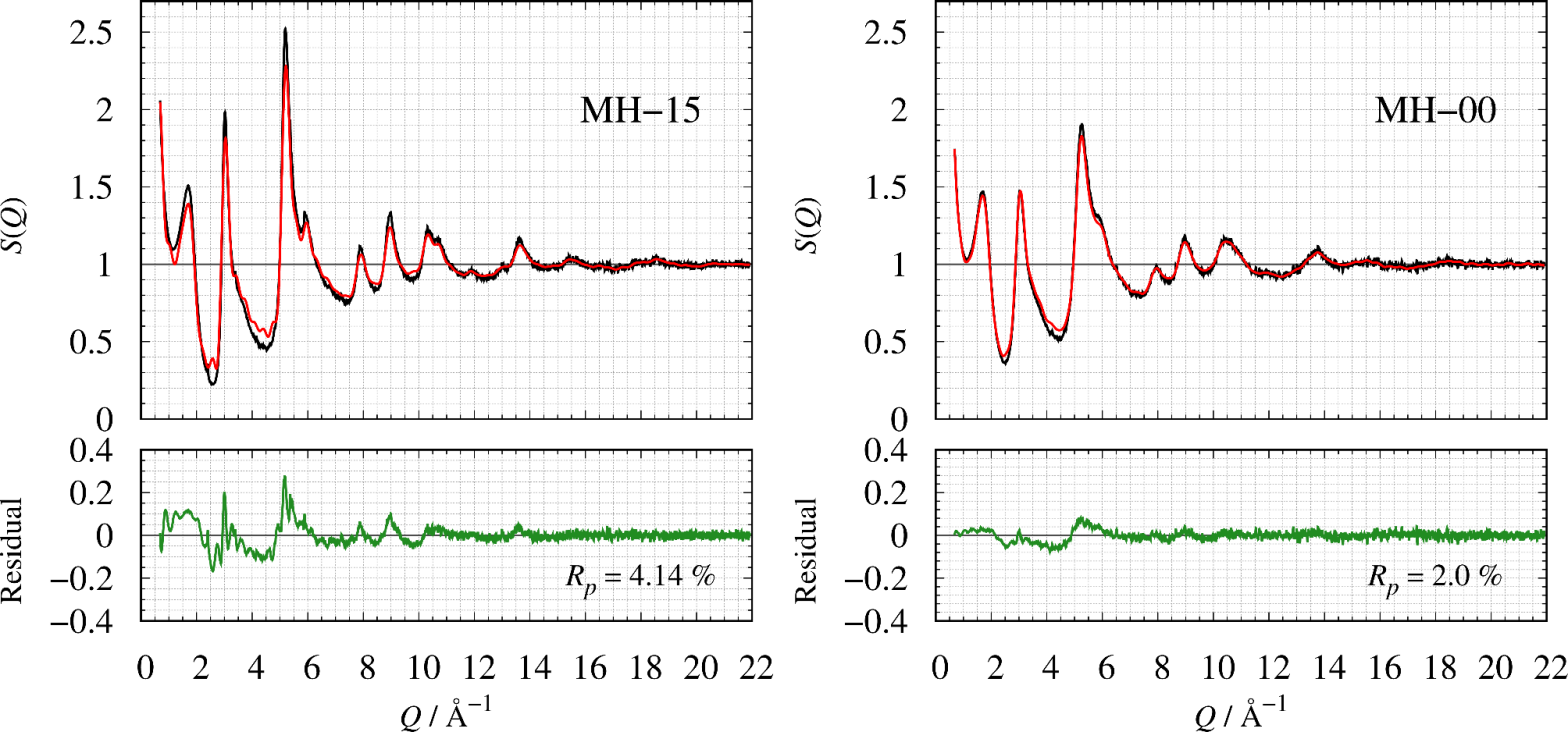
The comparison of *S*(*Q*) of MH-15 (left) and MH-00 (right). On the top panel, the red and the blue solid line shows the observed and calculated values, respectively. On the bottom panel, the green solid line is the corresponding residual curve.

**Fig. 4:**
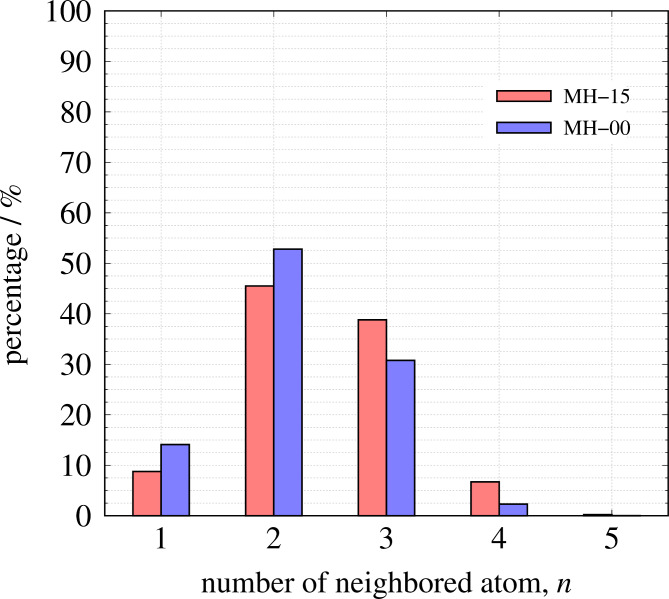
The histogram of the number of nearest-neighbor atoms whose distance is shorter than 1.8 Å.

**Fig. 5:**
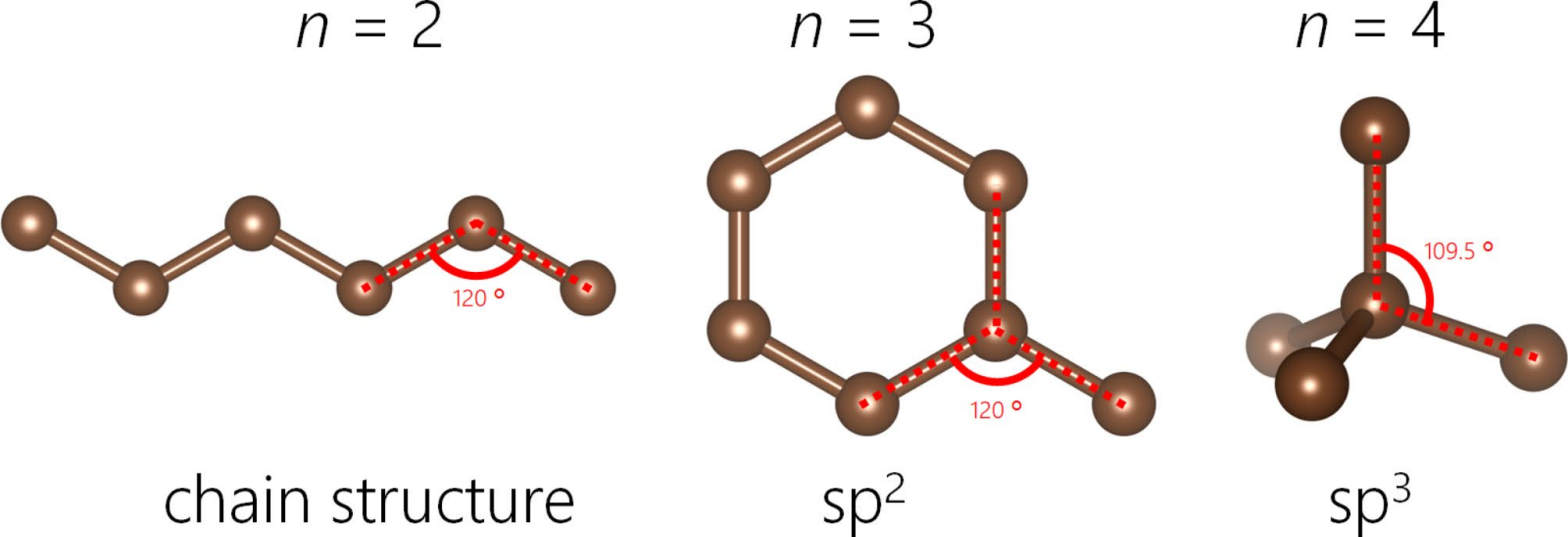
The structural model of each neighbor atom.

**Fig. 6:**
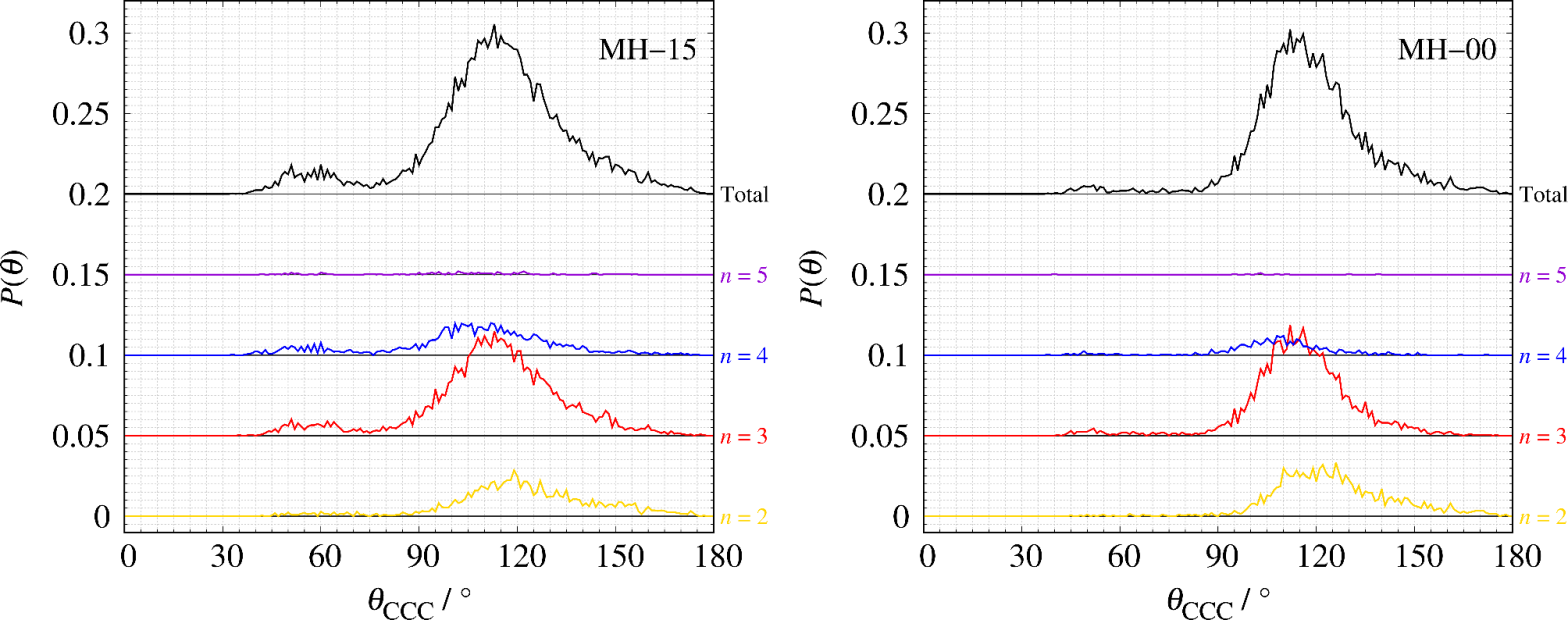
The angular histogram of the nearest-neighbor atoms classifying the number of atoms. Left: MH-15, right: MH-00.

**Fig. 7:**
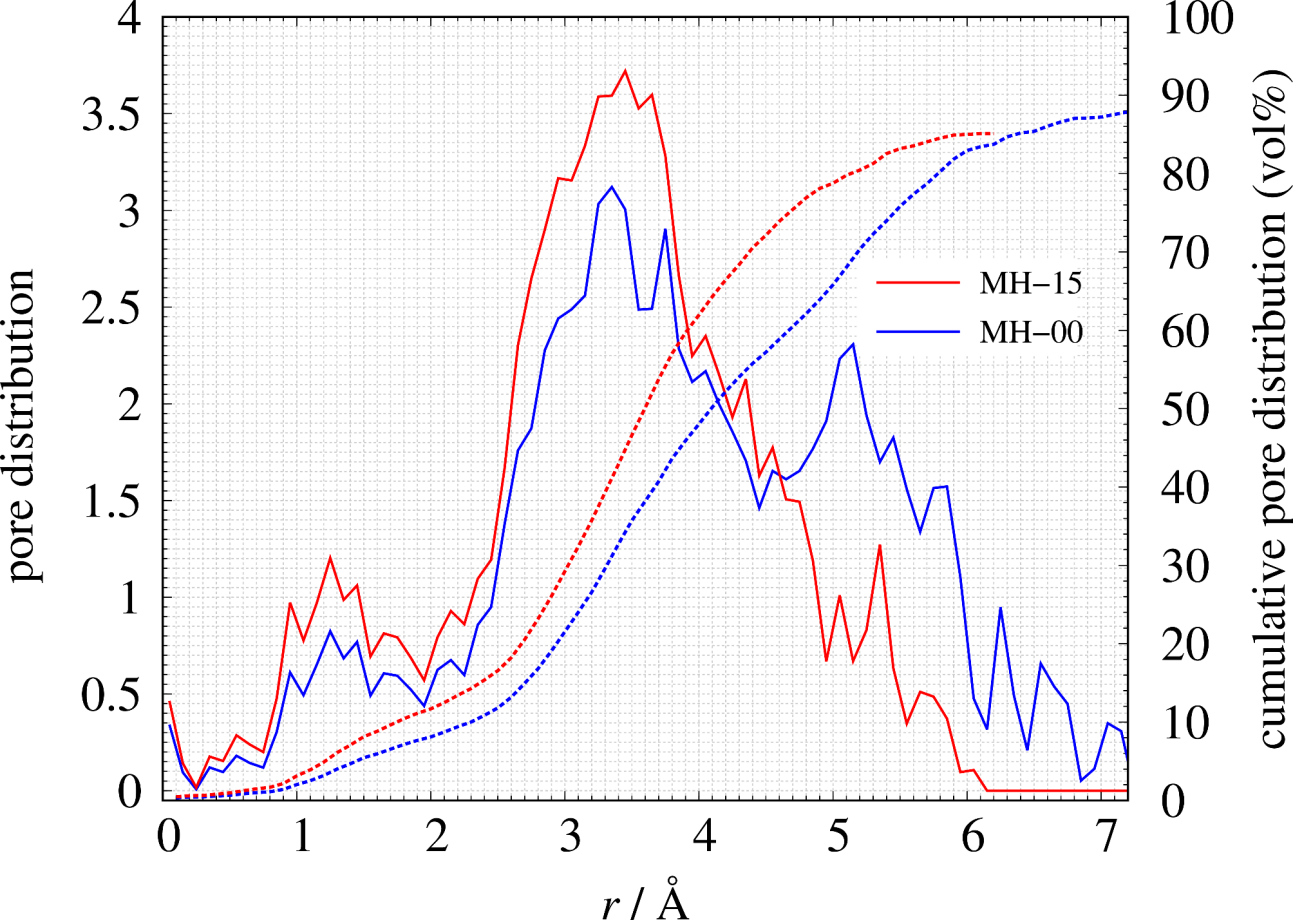
The comparison of pore distribution of MH-15(red) and MH-00 (blue). Solid line and broken line show a histogram of pore distribution and cumulative pore distribution, respectively.

Figure 3 shows the comparison of *S*(*Q*) between the observed value and the calculated value. The *R*-factor values *R*_p_ (defined in Eq. 6) of MH-15 and MH-00 are 4.14% and 2.0%, respectively. The nearest neighbor angular histogram (*r* ≤ 1.8 Å) of both specimens shown in Fig. S2 has a peak around *θ*_CCC_ = 120° and a small peak at *θ*_CCC_ = 60°. These results indicate that the RMC method that introduces the angular constraint term can construct a structural model that reproduces the experimental data while meeting physical constraints. Figure 4 shows the histogram of the numbers of nearest-neighbor atoms. *n* = 2, 3, and 4 are consistent with the chain structure, sp^2^ type structure, and sp^3^ type structure, respectively, as shown in Fig. 5. Both nearest-neighbor histograms have a maximum distribution at *n* = 2. As described in the Methods section, MH-15 and MH-00 samples are synthesized below the temperature at which graphitization occurred. It is noted that amorphous carbon remains the precursor character after heat treatment. In addition, MH-15 histogram has a greater ratio of *n* = 3, 4 than MH-00. The result shown in Fig. 4 is in good agreement with the carbon reaction state with heat treatment. Therefore, it is concluded that MH-15 develops a network structure between carbon atoms rather than MH-00 but cannot construct long-range ordered 6-member rings such as graphite. Figure 6 shows the nearest-neighbor angular histograms classified by the number of nearest-neighbor atoms. *n* = 2 histogram indicates the maximum peak around *θ*_CCC_ = 120°, which corresponds to the chain structure. *n* = 3 histogram also shows the maximum peak around *θ*_CCC_ = 120°, which indicates the hexagonal ring generation. *n* = 4 histogram has a peak around *θ*_CCC_ = 110°, which is consistent with the tetrahedral structure. Figure 7 shows the pore size histogram calculated from the constructed structural model using the local thickness algorithm^44^. The carbon atom radius is assumed to be *r* = 0.77 Å for this calculation. There are mainly two distributions in both curves. One pore shows a peak around *r* = 1.3 Å that corresponds to the hole located at the center of the 6-member ring. The others indicate a wide pore distribution above *r* = 2.0 Å. As mentioned above, both MH-15 and MH-00 were synthesized with heat treatment below the temperature at which graphitization occurred. Those results suggest that the wide pore distribution above *r* = 2.0 Å is formed between the bent carbon chain-like structure. The pore distribution of MH-15 shows 80% in small pores whose dimension is smaller than 5.0 Å. On the other hand, the pore distribution of MH-00 is slightly wider than that of MH-15 because the cumulative pore distribution shows 80% smaller than 5.8 Å.

**Fig. 8:**
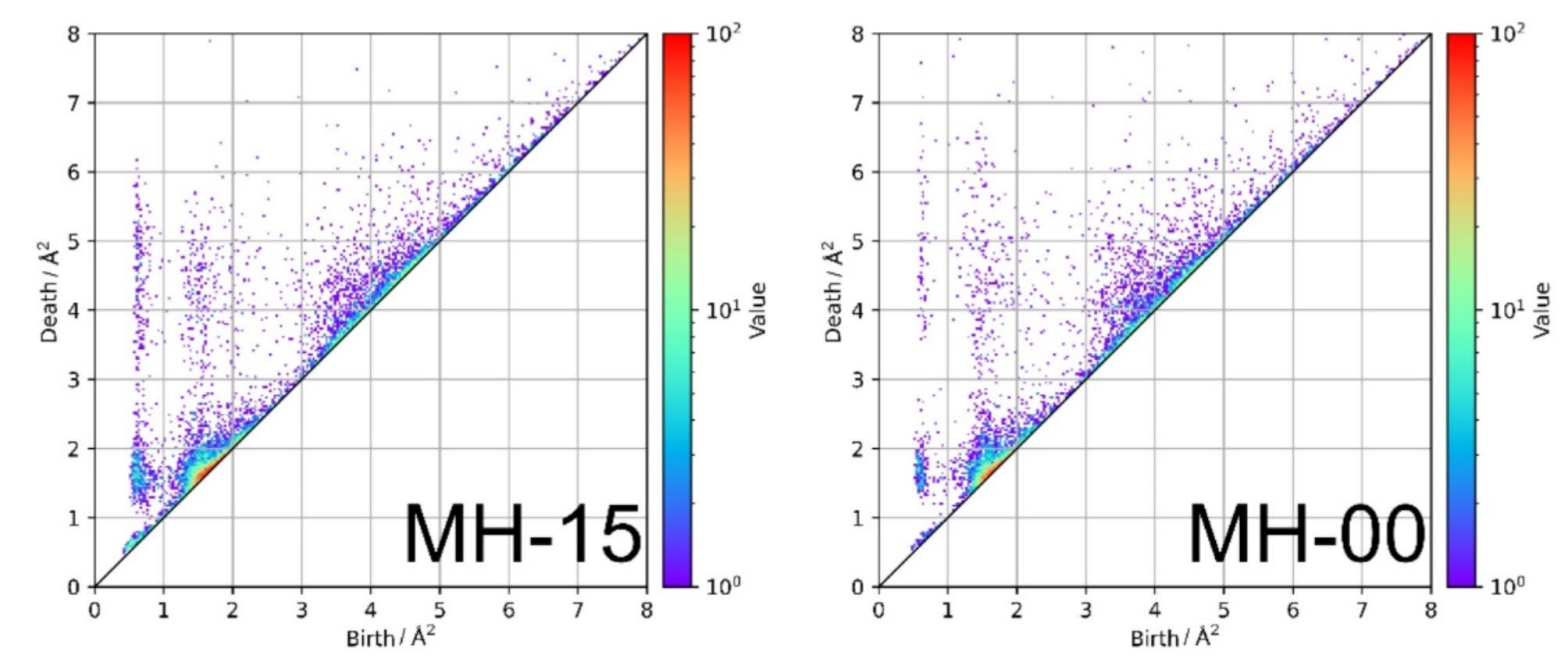
The comparison of PD1 diagrams between [0, 8]_birth_-[0, 8]_Death_ of MH-15 (left) and MH-00 (right).

**Fig. 9:**
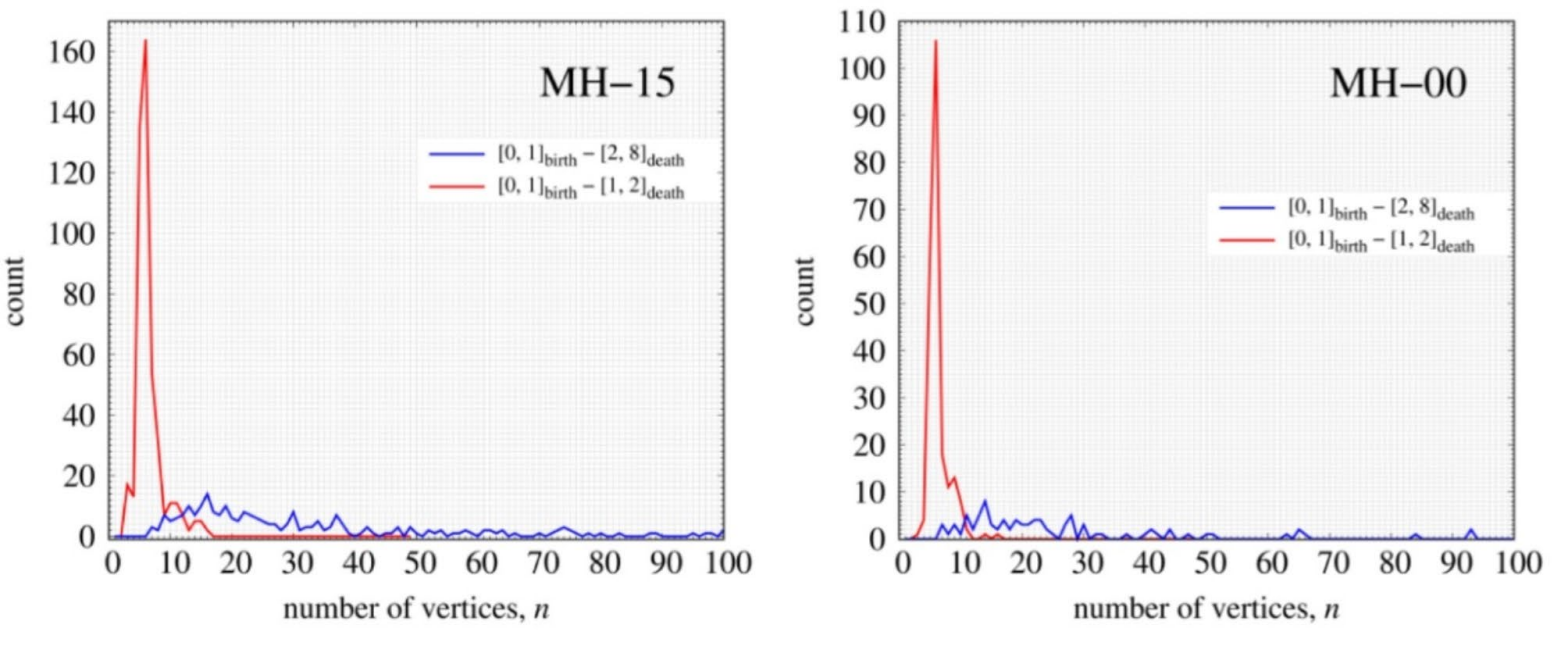
The histograms of the number of vertices are calculated for MH-15 (left) and MH-00 (right). Red solid line indicates the 6-member ring-like structure calculated between [0, 1]_birth_-[1, 2]_Death_ on PD1 diagram. Blue solid line indicates the large-sized rings calculated between [0, 1]_birth_-[2, 8]_Death_ on PD1 diagram.

**Fig. 10:**
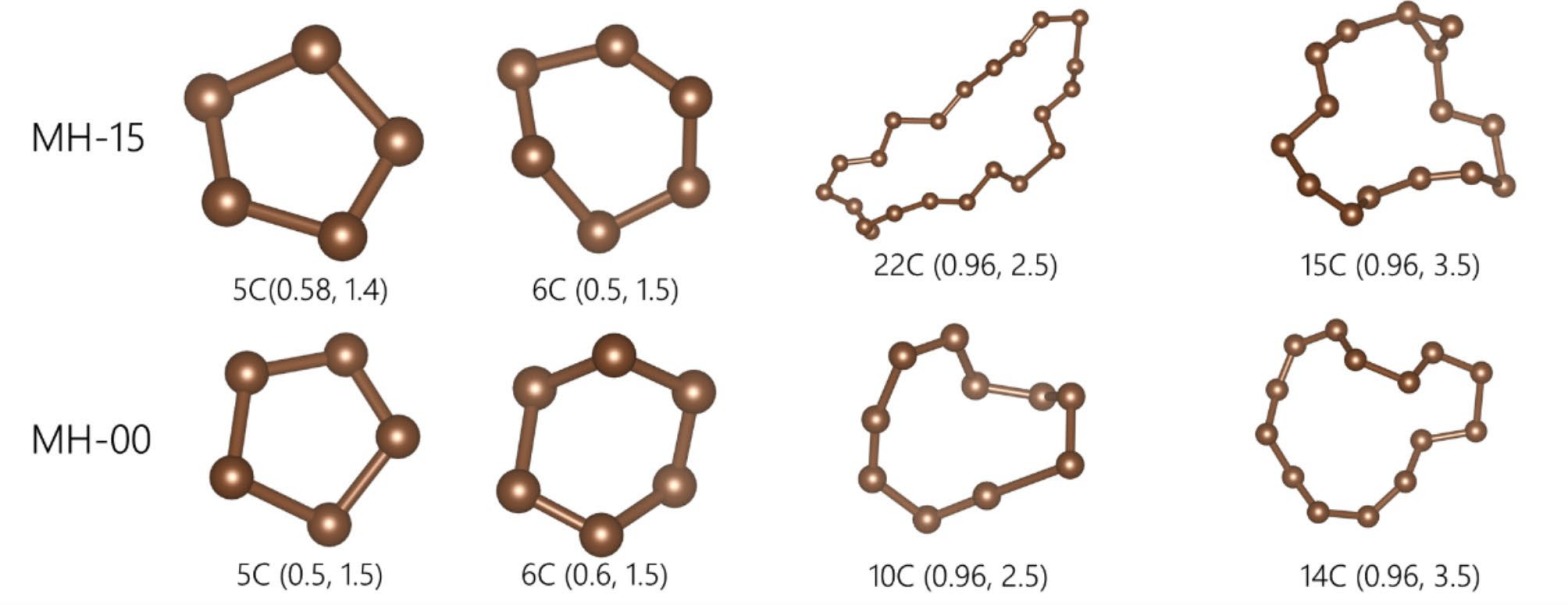
The ring structure was found by the persistent homology analysis of each sample: MH-15(top) and MH-00(bottom). The coordinate (birth, death) gives the birth and death values in PDs.

We performed persistent homology analysis on the estimated structural models of MH-15 and MH-00. PDs of MH-15 and MH-00 have a characteristic distribution between [0, 1]_Birth_ and [1, 8]_Death_ are shown in Fig. 8. To understand the relationship between birth-death pairs in PD1 and the local structure, the local structure is determined in two regions (i.e., [0, 1]_Birth_-[1, 2]_Death_ and [0, 1]_Birth_-[2, 8]_Death_) on PD1 by a stable volume^45^. Figure 9 shows histograms of the number of vertices in the local structure. Figure 10 also shows the typical corresponding ring structures. [0, 1]_Birth_-[1, 2]_Death_ and [0, 1]_Birth_-[2, 8]_Death_ corresponds to the low number of vertices and the large number of vertices, respectively. The histogram of the low-numbered vertices has a maximum at *n* = 6 which indicates the existence of 6-member rings shown in Fig. 10. The number of low-numbered vertices of MH-15 and MH-00 in the region is 464 and 225, respectively. The birth-death pairs [0, 1]_Birth_-[1, 2]_Death_ in PD1 directly relate to the number of *n*-member rings constructed with nearest-neighbored atoms. According to the results of the histogram of low-numbered vertices, heat treatment at high temperature accelerates the development of *n*-membered rings between the nearest-neighbored carbon atoms. On the other hand, the large-numbered vertices relate to the large rings forming inter-cluster networks, as shown in Fig. 10. The histogram of the large-numbered vertices has a wider distribution than that of the low-numbered vertices. The total amount of large-numbered vertices of MH-15 and MH-00 is 243 and 94, respectively. The histogram of MH-15 in Fig. 9 has a greater frequency between *n* = 10 and *n* = 20 than that of MH-00. According to the results of persistent homology analysis, the MH-15 meso-scale structure has well-developed carbon networks between *n*-member rings, while the MH-00 meso-scale structure mainly forms a chain-like structure left over from the precursor due to synthesis at *T* = 900 °C, which is too low to develop carbon networks.

## Conclusion

RMC modeling based on X-ray total scattering data for amorphous carbon materials enables the analysis of precise structural features, such as the nearest-neighbor coordination number and bond angle distribution. In addition, we have characterized micro-pore size distribution. The topological data analysis (persistent homology) suggests to us the relation between the number of networking carbon atoms with pore size. According to the result in this study, persistent homology analysis can easily evaluate the total amount of *n*-member rings and inter-cluster networks from an amorphous carbon structural model. From those, precise analysis tells the development of the local network structure with increasing heat treatment temperature. Differences in the pore size distribution and carbon network structure can indicate differences in physical/chemical characteristics of carbon materials. The pore size distribution calculated from RMC suggests that the pore located above 2.0 Å is related to the storage amounts of Li-ions and small molecular energy gases. The result is consistent with the nitrogen adsorption study^46^. It is well known that amorphous carbon constructs a hierarchical structure between atomistic and nm-ordered pores. In the next step, we will perform small angle X-ray scattering combined with RMC^47^ to analyze nm-sized pore structures that also play an important role in the storage characteristics of Li-ions and energy gases. These comprehensive studies provide a more precise relation between atomic and nanometer scale structure with physical/chemical characteristics of amorphous carbon materials.

## Methods

### Sample Preparation

The two amorphous carbon samples that are named CNovel^®^ were purchased from Toyo Tanso Co., Ltd. Two amorphous carbon materials were prepared by the template method. Carbon was converted by heat treatment to an organic magnesium salt at *T* = 900 °C under N_2_ flow as described in the following report^46^. The MgO was dissolved by H_2_SO_4_ treatment. After washing with water and air-dried, the MgO templated carbon labeled MH-00 below was synthesized. Heat treatment was performed on MH-00 at *T* = 1500 °C for 1 h under Ar atmosphere. Finaly, MgO templated carbon with the heat treatment is synthesized, labeled MH-15 below.

## X-ray total scattering measurement

The amorphous carbon was sealed in a borosilicate glass capillary whose diameter is 2.0 mm. The blank capillary profile was used as background data. We performed total scattering measurements using a SmartLab (Rigaku Corp.) equipped with a high-speed 1D detector. The X-ray energy is *E*_Ag Kα_ = 22.11 keV, which was monochromatized by an elliptic *d*-space graded multilayer mirror focused on the detector position (Rigaku Corp.). The total scattering measurement was performed with the scattering angle 2*θ* and its corresponding scattering vector *Q* ranging from 2.5° and 0.489 Å^−1^ to 156° and 21.918 Å^−1^, respectively. The structure factor *S*_obs_(*Q*) and the pair distribution function *G*_obs_(*r*) were obtained using established correction procedures^43,48–53^.

## RMC calculation

The basic RMC algorithm reported by R. L. McGreevy and L. Pusztai constructs a structural model whose calculated structure factor *S*(*Q*) and pair distribution function *G*(*r*) are consistent with the experimental value^54^. General simulation techniques including MD treat the atomic (particle) arrangement as a two-body problem (i.e., pair potential). For amorphous solids (e.g., amorphous silicon, porous carbon materials), it is widely known that the pair potential cannot construct the appropriate structural model due to the fact that more than three-body covalent interactions are critical. Therefore, many researchers have reported that the potential term implementing an angular constraint effect eliminates the unphysical atomic arrangement and then can provide the structural model to discuss the physical property between experimental and calculation values^55–57^. In this study, we tried an angular constraint term^57^ that eliminates un-physical atomic arrangement as a cost function of the RMC method as below:

2 $$\:\begin{array}{c}f\left(\text{cos}{\theta}_{ijk}\right)={\left(\text{cos}{\theta}_{ijk}-\text{cos}\frac{2}{3}\pi\:\right)}^{2}w\left({r}_{ij}\right)w\left({r}_{ik}\right)\end{array}$$

where $\:{\theta\:}_{ijk}$ is the bond angle of the neighbor carbon atoms within $\:{r}_{ij}$ and $\:{r}_{ik}$, $\:w\left(r\right)$ is the weight function defined in Eq. 3.

3 $$\:\begin{array}{c}w\left({r}_{ij}\right)=\left\{\begin{array}{c}1.0\:for\:r<{r}_{\text{c}\text{u}\text{t}\text{o}\text{f}\text{f}}-2\varDelta\:r\\\:0.5\left(1+\text{cos}\left(\frac{\pi\:}{2}\frac{\left({r}_{ij}-{r}_{\text{c}\text{u}\text{t}\text{o}\text{f}\text{f}}+{\Delta\:}r\right)}{{\Delta\:}r}+1\right)\right)for\:{r}_{\text{c}\text{u}\text{t}\text{o}\text{f}\text{f}}-2\varDelta\:r\le\:r<{r}_{\text{c}\text{u}\text{t}\text{o}\text{f}\text{f}}\\\:0\:for\:r\ge\:{r}_{\text{c}\text{u}\text{t}\text{o}\text{f}\text{f}}\end{array}\right.\end{array}$$

where $\:{\Delta\:}r$ is the step size (0.1 Å in this study), $\:{r}_{\text{c}\text{u}\text{t}\text{o}\text{f}\text{f}}$ is the cutoff distance which is randomly generated between $\:{r}_{c}-{\Delta\:}r$ and $\:{r}_{c}+{\Delta\:}r$ whose center $\:{r}_{c}$ is 1.6 Å.

When RMC generates a new arrangement in a certain trial, the cost $\:\beta\:$ is calculated between the center neighbor *j*-th and *k*-th atoms within $\:{r}_{\text{c}\text{u}\text{t}\text{o}\text{f}\text{f}}$ as below:

4 $$\:\begin{aligned}\beta\:=\text{exp}\left(-\sum_{j}^{n}\sum_{k\ne j}^{n}f\left({\theta}_{\text{c}\text{e}\text{n}\text{t}\text{e}\text{r}jk}\right)\right).\end{aligned}$$

Then, the RMC evaluates whether the new arrangement can be generated or not using the Metropolis method with the random number $\:\xi\:$ between 0 and 1 as below:

5 $$\:\begin{array}{c}\begin{array}{c}reject,\:for\:\xi\:>\beta\:,\\\:accept,for\:otherwise.\end{array}\end{array}$$

The simulation cell size was 40 × 40 × 40 Å^3^ with periodic boundary conditions. There are 4910 and 4011 C atoms in the simulation cells of MH-15 and MH-00. The detailed calculation condition is listed in Table 1. The RMC only uses a movement trial and the maximum movement distance is 0.2 Å for each MC step. The estimated structural model was evaluated using the *R*-factor *R*_*p*_ shown in Eq. 6:

6 $$\:\begin{array}{c}{R}_{p}=\sqrt{\frac{{\sum}_{i}{\left({S}_{\text{o}\text{b}\text{s}}\left({Q}_{i}\right)-{S}_{\text{R}\text{M}\text{C}}\left({Q}_{i}\right)\right)}^{2}}{{\sum}_{i}\left({S}_{\text{o}\text{b}\text{s}}{\left({Q}_{i}\right)}^{2}\right)}}\end{array}$$

The RMC trial is performed to decrease *R*_*p*_ by changing atomic configurations in the simulation box until *R*_*p*_ becomes almost constant.

### Persistent homology analysis

The HomCloud code^40^ was used for PH analysis of the amorphous carbon structural model constructed by the present RMC. We focused on the rectangular region that was derived into a 256 × 256 mesh, between [0.0, 8.0]_birth_ and [0.0, 8.0]_Death_ in PD1.

## Electronic supplementary material

Below is the link to the electronic supplementary material.

Supplementary Material 1

## Data Availability

The datasets used during the current study available from the corresponding author on reasonable request.
