# Supplementary material for: Local structure of Amorphous carbon investigated by X-ray total scattering and RMC modeling
Source: Sci Rep. 2024 Oct 25;14:25298. doi: 10.1038/s41598-024-76796-x (PMC11511976; doi:10.1038/s41598-024-76796-x)
Supplement: Supplementary file 1 — Supplementary Material 1 [file 41598_2024_76796_MOESM1_ESM.docx]

**Figures**


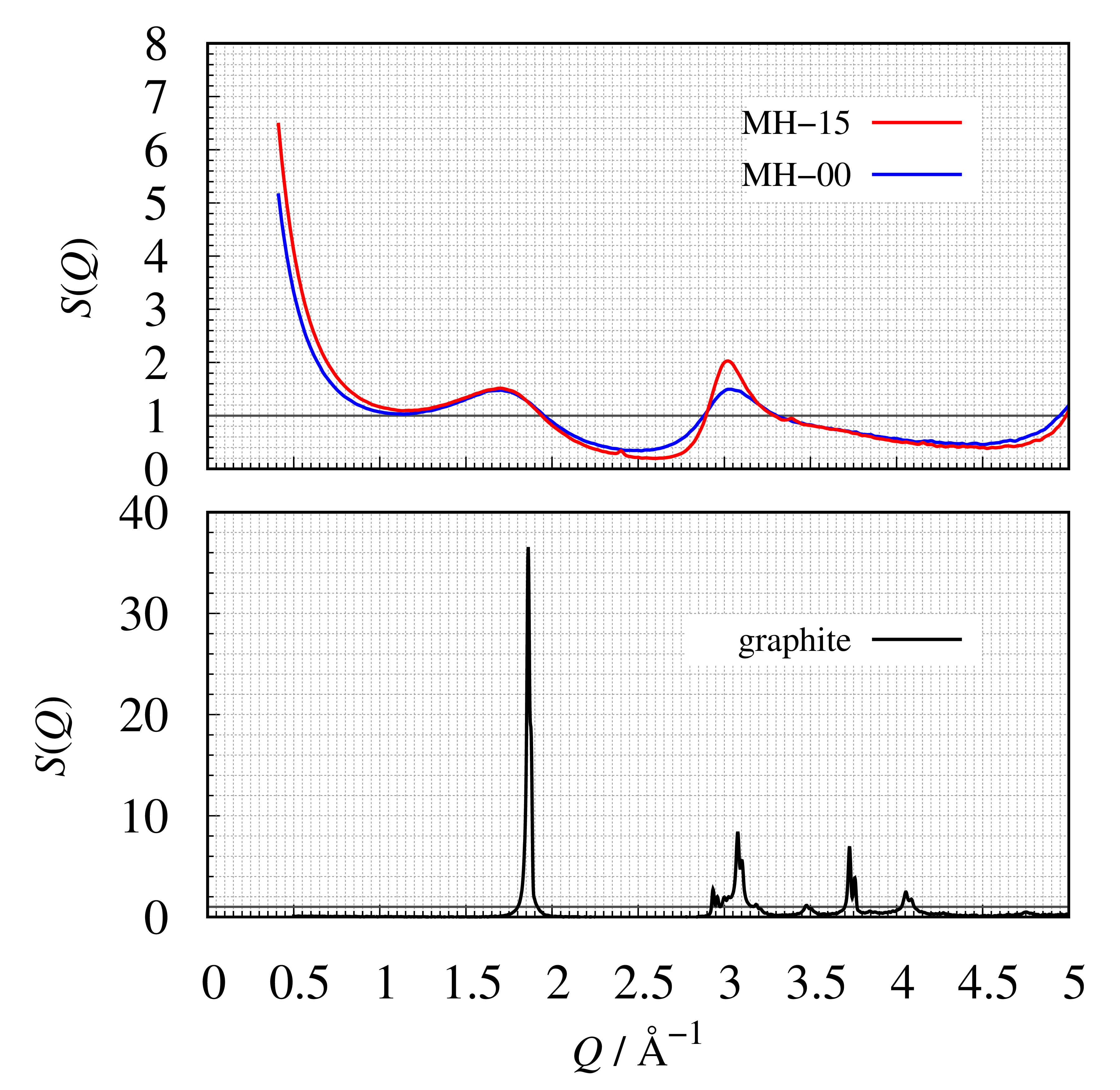


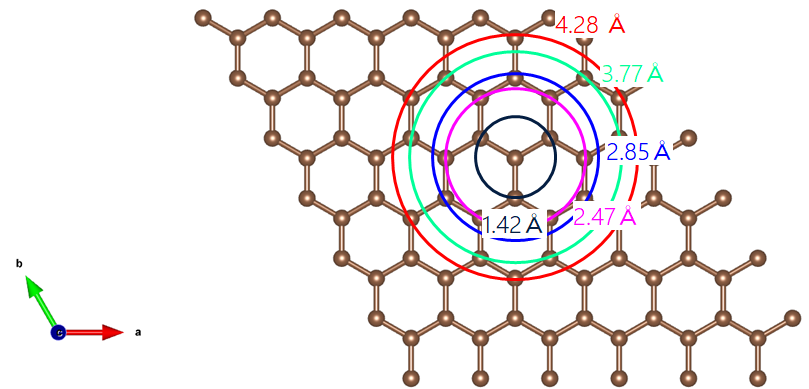

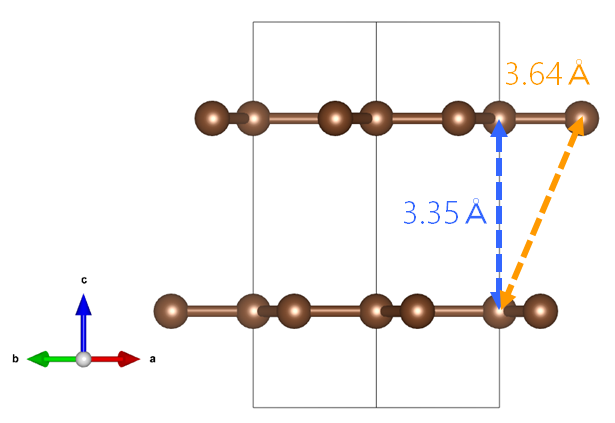


Figure S1. The comparison of *S*(*Q*) of MH-15, MH-00 and graphite. The top panel shows *S*(*Q*) of MH-15(red) and MH-00(blue). The middle panel shows graphite *S*(*Q*). The bottom illustrations show the typical graphite structures with indicating characteristic distances between carbon atoms. The first peak of graphite *S*(*Q*) shown in the middle corresponds to the interlayer distance of *d* = 3.35 Å and *Q* = 1.874 Å^-1^.


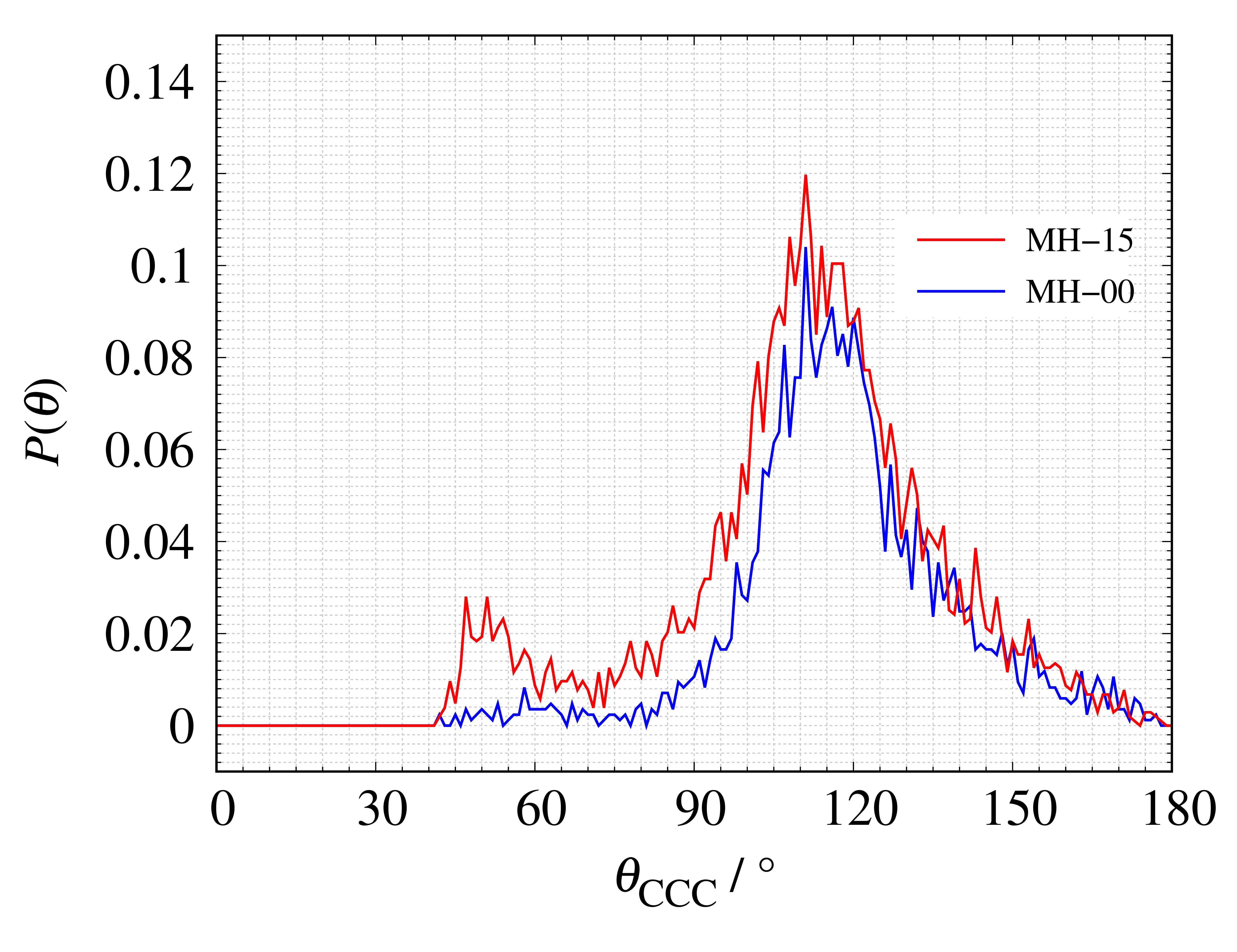


Figure S2. The angular histogram between the nearest-neighbor carbon atoms (*r* ≤ 1.8 Å).
